# Vineyard Management and Physicochemical Parameters of Soil Affect Native *Trichoderma* Populations, Sources of Biocontrol Agents against *Phaeoacremonium minimum*

**DOI:** 10.3390/plants12040887

**Published:** 2023-02-16

**Authors:** Guzmán Carro-Huerga, Sara Mayo-Prieto, Álvaro Rodríguez-González, Rosa E. Cardoza, Santiago Gutiérrez, Pedro A. Casquero

**Affiliations:** 1 Grupo Universitario de Investigación en Ingeniería y Agricultura Sostenible (GUIIAS), Instituto de Medio Ambiente, Recursos Naturales y Biodiversidad, Universidad de León, Avenida Portugal 41, 24071 León, Spain; 2 Grupo Universitario de Investigación en Ingeniería y Agricultura Sostenible (GUIIAS), Área de Microbiología, Escuela de Ingeniería Agraria y Forestal, Campus de Ponferrada, Universidad de León, Avenida Astorga s/n, 24400 Ponferrada, Spain

**Keywords:** biological control, soil analysis, principal component analysis, multiple factor analysis, *in vitro* assays, grapevine trunk diseases

## Abstract

Native strains of *Trichoderma* in vineyard soil represent an opportunity for reducing the incidence of grapevine trunk diseases (GTDs) in vineyards. Moreover, its relationship with the environment (physicochemical soil characteristics and farming management practices) remains unclear. In the current study, a survey was carried out on farming management used by viticulturists, and soil samples were studied to analyze their physicochemical properties and to isolate *Trichoderma* strains. Later, statistical analyses were performed to identify possible correlations between *Trichoderma* populations, soil management and soil characteristics. In addition, *in vitro* tests, including antibiosis and mycoparasitism, were performed to select those *Trichoderma* strains able to antagonize *Phaeoacremonium minimum*. In this study a positive correlation was found between the iron content and pH in the soil, and a lower pH increases *Trichoderma* populations in soils. Vineyard management also affects *Trichoderma* populations in the soil, negatively in the case of fertilization and tillage and positively in the case of herbicide spraying. Two *Trichoderma* native strains were selected as potential biocontrol agents (*Trichoderma gamsii* T065 and *Trichoderma harzianum* T087) using antibiosis and mycoparasitism as mechanisms of action. These results led to the conclusion that native *Trichoderma* strains hold great potential as biological control agents and as producers of secondary metabolites.

## 1. Introduction

Biological control agents (BCAs) are important instruments to reduce pests and diseases with the aim of protecting crops. They are defined as living agents that constantly target pests and can be classified into four different categories: whether they are added for permanent or temporary establishment, and whether they are used with or without targeted human intervention [1]. Moreover, antimicrobial secondary metabolites produced by agriculturally important fungi can also be used as a green solution to protect crops. They are low-molecular-mass metabolites produced during the late growth phase and are divided into different biosynthetic categories such as metabolites derived from shikimic acid, those derived from amino acids, those derived from acetyl-CoA and those derived from sugars [2]. These strategies would prevent the use of synthetic pesticides or reduce their use, in line with ongoing European Union proposals [3].

The *Trichoderma* genus contains BCAs that are mainly used in biocontrol for agricultural purposes due to their versatility and capability of adaptation [4]. More than 375 species have been described [5] and this fungus is one of the most bio-based products worldwide [6]. As BCAs, *Trichoderma* strains can display a great variety of biocontrol mechanisms, such as mycoparasitism, lytic enzymes, antibiosis, secondary metabolites, competition with pathogens and the soil microbial community, plant root colonization and the induction of plant defense responses [7].

*Vitis vinifera* L. (grapevine plant) cultivars are the most widely planted in the world and require high production costs, of which one of the most important is associated with pest and disease management. Nowadays the most significant destructive diseases in viticulture are grapevine trunk diseases (GTDs) [8]. A sustainable and eco-efficient approach is needed to solve this problem [9]. Furthermore, *Trichoderma* strains are among the most widely tested biological control agents against fungi involved in GTDs [10]. However, *Trichoderma* results after field trials are uncertain and still show great variability and disparity [11,12]. In the current study, first, we try to elucidate the factors that could favor the persistence of *Trichoderma* in the soil in order to protect the roots of vine plants. Farming management practices in soils are an important source of variation in ecosystems because they modify microbial composition, including *Trichoderma*, especially in vineyards [13,14,15,16,17,18]. Therefore, a survey of the main farming management practices was performed in this research to give us information about their influence on soil *Trichoderma* populations. Herbicides are important tools for weed management and their use is increasing worldwide. In addition, they can alter the soil microbiome and nutrient composition of grapevine plants [18]. Thus, surveys on the use (or not) of herbicides in rows of vineyards could show a correlation between soil health and *Trichoderma* populations. In addition, tillage intensity can modify the soil population of microorganisms such as *Trichoderma* spp., Actinomycetes and *Gliocladium* spp. [19]. In addition, fertilizers such as manure or chemical fertilizers could influence the microbial population [20,21]. Finally, the main physicochemical nutrients present in the soil have been shown to be an important source of variation in fungal and bacterial communities in soil vineyards [19,22,23]. Thus, all of these variables will be evaluated in our research.

Another point to evaluate is the use of indigenous *Trichoderma* from the soil to protect grapevine plants against one of the most prevalent fungi involved in GTDs, *Phaeoacremonium minimum*. This pathogen is the pioneer fungus of Petri, Esca and grapevine leaf stripe diseases [24]. Not only can it colonize woody tissues [24] but is also a soilborne pathogen [25]. Previous studies have shown that there are native *Trichoderma* strains isolated from soils or grapevine plants that can protect grapevine plants from GTDs. In South Africa, for example, two strains (*T. atroviride* T-77 and *T. atroviride* USPP T1) can colonize the roots of grapevine plants and activate the host defense system of the plant [26]. Another study showed that after testing 16 *Trichoderma* isolates from Southern Italy, some of them protect grapevine plants against a great number of fungal pathogens that cause GTDs [27]. Moreover, native *Trichoderma* strains have been isolated from vineyards in British Columbia, Canada, as effective biocontrol agents against Botryosphaeria dieback [28]. In Spain, strains of *T. harzianum* had positive results after their application in young vine plants [29].

In this study we analyzed the following hypotheses: (i) soil physicochemical properties and farming management practices affect native *Trichoderma* populations, and (ii) *Trichoderma* native strains isolated from vineyard soils are able to control *P. minimum*. The specific objectives are to (1) isolate *Trichoderma* strains from the soil, (2) identify the relationship between the physicochemical soil characteristics and the abundance of *Trichoderma* and (3) identify *Trichoderma* strains for biocontrol of *P. minimum* in the soil.

## 2. Results

### 2.1. Study Sites and Management

Ten plots were evaluated in order to isolate native *Trichoderma* strains. Each of these plots were codified using three to four letters that refer to the location. There are four plots (PLC, RIB, CAL and MAZ) belonging to Protected Designation of Origin (PDO) León; one plot (PEGB) to PDO Toro; another three plots (GLM, GLG and GVG) to PDO Bierzo; and two plots (ARE and TER) from PDO Ribera del Duero. Data recorded in Table 1 refer to the type of management and are represented in a dichotomous format (yes/no) to identify if viticulturists tilled the rows, sprayed herbicides in the rows or applied fertilizer (manure or chemical).

### 2.2. Soil Physichochemical Parameters

Ten plots were selected to isolate *Trichoderma* strains (Table 2). The pH ranged from 5.12 in PEGB up to 8.26 in TER. The texture was also evaluated, and it was represented as the percentage of the different textural sizes (sand, silt and clay). The percentage of organic matter was also identified as SOM (soil organic matter). Finally, regarding to the nutrients, values of total nitrogen (total N), phosphorus (P), calcium (Ca), magnesium (Mg), potassium (K), manganese (Mn), iron (Fe), copper (Cu), zinc (Zn) and boron (B) were also determined (Table 2).

### 2.3. Isolation of Trichoderma Strains from Soil Samples. Selection, Genus Assigment and Quantification

**Figure 1:**
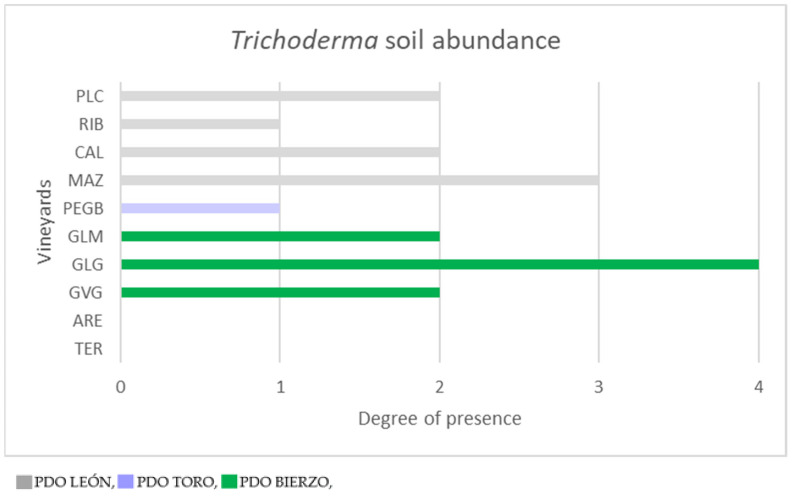
Presence of *Trichoderma* spp. in vineyard soils. Values are assigned as described in Section 4.3.2; values range from 0 to 4 according to the serial dilution to which they were isolated (10^−3^ = 4: 10^−2^ = 3; 10^−1^ = 2; 1 = 1; none = 0). Plot locations: PLC (La Antigua, Castilla y León); RIB (La Bañeza, Castilla y León); CAL (Gordoncillo, Castilla y León); MAZ (Gordoncillo, Castilla y León); PEGB (El Pego, Castilla y León); GLM (Cacabelos, Castilla y León); GLG (Cacabelos, Castilla y León); GVG (Cacabelos, Castilla y León); ARE (Peñafiel, Castilla y León); TER (Peñafiel, Castilla y León).

First, *Trichoderma* spp. isolates were morphologically identified according to Rifai 1969 [30]. Then they were selected to avoid duplication of the same isolates in the same plots due to their morphological characteristics, and finally they were confirmed using genetic identification through PCR amplification using the primer pair ITS4-ITS5 and using amplicon sequencing. Thus, a total of 21 *Trichoderma* isolates were identified according to different phenotypes selected from vineyards (Supplementary Figure S1). In PDO LEON, eight isolates were selected that are described as follows: T099 was selected from plot PLC and was found up to 10^−1^ in serial dilution, so a value of 3 was assigned to this vineyard in terms of quantity; strains T181 and T182 were isolated from RIB up to the original undiluted concentration, so a value of 1 was assigned; strains T063, T064 and T065 were identified in plot CAL up to a concentration of 10^−1^, so a value of 2 was given to this plot; and T066 and T067 from plot MAZ were selected up to a concentration of 10^−2^, so a value of 3 for quantification of *Trichoderma* was given. In PDO TORO, a single strain (T087) was isolated from plot PEGB and a concentration without dilution was found. Thus, a value of 1 was determined for this plot. In PDO BIERZO, twelve strains were isolated according to the following plots: three strains (T186, T187 and T188) from plot GLM up to a concentration of 10^−1^, giving a value of 2 to this plot; six strains (T194, T195, T196, T197, T198 and T199) from plot GLG with the highest value for quantification assigned as a 4 due to isolates found in a concentration of up to 10^−3^; and another three strains (T183, T184 and T185) from plot GVG with a quantification value of 2. No *Trichoderma* strains were isolated from the soils of PDO RIBERA DEL DUERO where two plots were surveyed (ARE and TER) so that a value of zero was assigned to these two plots. Representation of these values can be visualized in Figure 1.

### 2.4. Parameters Involved in Trichoderma Abundance

#### 2.4.1. Identification of Parameters Involved in *Trichoderma* spp. Abundance and Physicochemical Parameters

**Figure 2:**
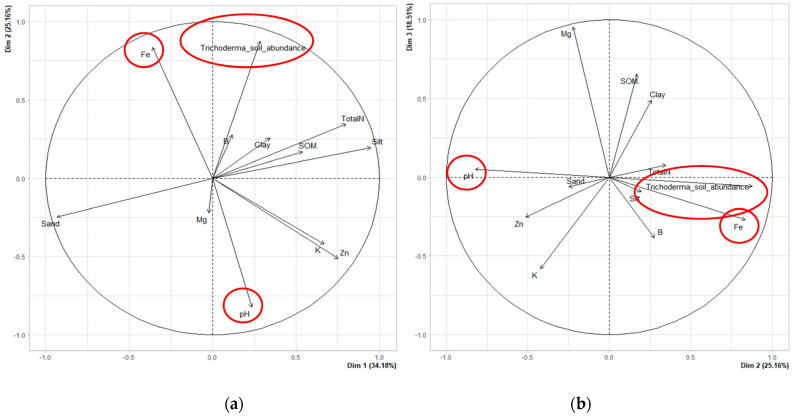
Principal components analysis (PCA) of analyzed soils: (**a**) Comparison between dimensions 1 and 2; (**b**) Comparison between dimensions 1 and 3. Soil physicochemical analysis parameters: pH of soil; percentage of clay, sand and silt; soil organic matter (SOM), nitrogen (Total N); assimilable phosphorus (P); the cations potassium (K), calcium (Ca) and magnesium (Mg); and the microelements manganese (Mn), iron (Fe), copper (Cu), zinc (Zn) and boron (B). Red circles indicate parameters that are significant between them.

PCA analyses were performed to evaluate the correlation between data of the physicochemical properties of the soil among the sixteen soil parameters and the *Trichoderma* soil abundance. First, they were statistically analyzed to evaluate if a normal distribution was identified and if there was no collinearity between them. Then 12 parameters were chosen for this analysis (Table 3). For the PCA analysis that was carried out on the twelve parameters evaluated, the first three dimensions were considered. The characteristics of each dimension were determined based on the estimated factor loadings. In this case, 77.48% of the information (variance) contained in the data were retained by the first three principal components (Dim1 = dimension 1, Dim2 = dimension 2, Dim3 = dimension 3). Dim1 comprised 34.18% of the total variation, while Dim2 and Dim3 explained 25.16% and 18.14%, respectively (Figure 2). According to the aim of this research, the essential parameter was *Trichoderma* soil abundance. Thus, dimension 2 was where a significant correlation was achieved. Dim2 was positively correlated with the presence of *Trichoderma* soil abundance and Fe, but negatively correlated with the pH value (Figure 2). This dimension revealed that pH value had a correlation value of −0.8223, Fe parameter, 0.8345 and *Trichoderma* soil abundance, 0.8761. In Table 4, the parameters that revealed a significant correlation in dimension 2 are represented [p value (*p* < 0.05)], including the *Trichoderma* soil abundance, pH and Fe. No significant clusters were identified in the clustering analysis or graphical analysis of individual plots.

#### 2.4.2. Identification of Parameters Involved in *Trichoderma* spp. Abundance and Management Regime

**Figure 3:**
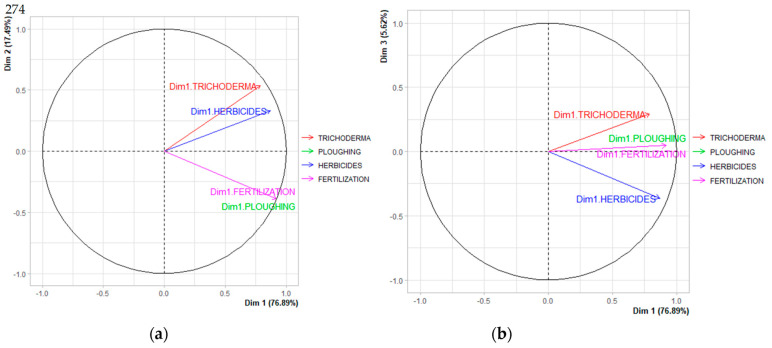
Multiple factor analysis (MFA) of analyzed soils: (**a**) Comparison between dimensions 1 and 2; (**b**) Comparison between dimensions 1 and 3.

MFA analysis was carried out to evaluate the management regime and *Trichoderma* soil abundance where datasets are structured in two sets of variables (continuous or categorical) and studied simultaneously. One quantitative component (*Trichoderma* soil abundance) was compared to three qualitative components (tillage, herbicide and fertilization). After normality and collinearity, an MFA was performed. The MFA analysis was carried out among the four parameters, and the first three dimensions were considered. The characteristics of each dimension were determined based on the estimated factor loadings. In this case, 100.00% of the information (variance) contained in the data were retained by the first three main components (Dim1 = dimension 1, Dim2 = dimension 2, Dim3 = dimension 3). Dim1 comprised 76.89% of the total variation, while Dim2 and Dim3 explained 17.49% and 5.62%, respectively (Figure 3). According to the aim of this research, the essential parameter is *Trichoderma* soil abundance, so that the dimension where a significant correlation was achieved was in dimension 1. Dim1 showed a significant level of correlation with the presence of *Trichoderma* soil abundance (0.7903), tillage (0.9207), herbicides (0.8694) and fertilization (0.9207) (Table 5). Dim2 revealed a pH value with a correlation value of −0.8223, Fe parameter with 0.8345 and *Trichoderma* soil abundance with 0.8761. In Table 6, parameters that revealed a significant correlation in dimension 1 are represented and *p* values (*p* < 0.05) are included. In Table 6, a significant positive correlation is achieved by the parameter *Trichoderma* soil abundance (0.7903 and *p* value = 0.065) and no fertilization (*p* value = 0.0002), no tillage in lines (*p* value = 0.0011) and spraying of herbicides in line (*p* value = 0.0002). No significant clusters were identified in the clustering analysis or graphical analysis of individual plots.

### 2.5. Trichoderma Molecular Characterization

The *Trichoderma* strains T065 and T087 were molecularly identified to the species level. In the case of T087, sequences of the ITS region and an internal region of the *tef1*a gene were used for phylogenetic and identification analysis. The T065 strain was identified using 20 housekeeping genes as described by [31]. Based on these sequences, these two strains were identified as *T. gamsii* T065 and *T. harzianum* T087.

### 2.6. In Vitro Antifungal Assays

#### 2.6.1. Membrane Assays

**Figure 4:**
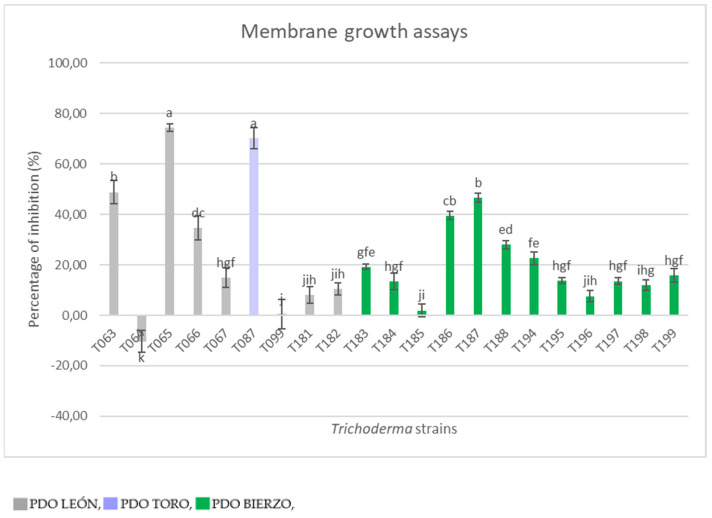
Antifungal membrane assay of *Trichoderma* spp. isolates after 14 days at 25 °C against the plant pathogenic fungus *P. minimum* on PDA.Results are expressed as the inhibition percentage compared to the control *P. minimum* grown alone. Values are means of four replicates. The letters indicate means among which there are no statistically significant differences (*p* = 0.05), according to the Duncan post hoc test applied to normalized data.

The biocontrol potential among the *Trichoderma* strains isolated from soil based on their ability to produce metabolites that may inhibit *P. minimum* revealed a great variability of results even from isolates that were identified from the same soil (Figure 4). *Trichoderma* isolates T065 and T087 inhibited *P. minimum* growth by more than 70% with the highest value of inhibition found for T065 (74.32%). T063, T066, T186, T187 and T188 also showed an important rate of inhibition values (25–50%). Most strains T067, T181, T182, T183, T184, T194, T195, T196, T197, T198 and T199 exhibited a moderate to low inhibition of the pathogen (5–25%). Finally, T099 and T064 revealed a nearly zero or negative capacity for inhibition with the lowest value of −10.35% (T099).

#### 2.6.2. Antifungal Dual-Culture Assays

**Figure 5:**
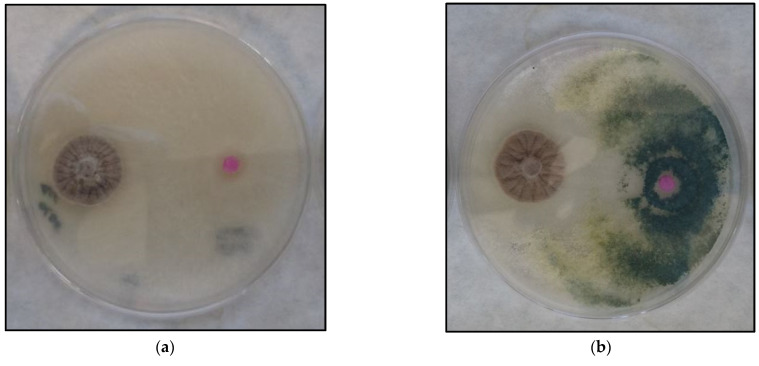
Dual-confrontation assay against *P. minimum* after five days of inoculation: (**a**) *Trichoderma* isolate T065; (**b**) *Trichoderma* isolate T087. *Trichoderma* and *P. minimum* growth can be observed at the right and left sides of each plate, respectively.

A direct confrontation assay was performed with the two *Trichoderma* strains exhibiting the highest values in membrane assays (T065 and T087). Both were able to inhibit the growth of *P. minimum* (Table 7). There were no significant differences among them related to *P. minimum* growth inhibition rate. No production of yellow pigment and sporulation on pathogen was identified but there was a significant difference between them in terms of sporulation on plate. A high production of spores was identified in T087 but no spore production was seen after 5 days in T065 (Figure 5).

## 3. Discussion

This study investigated the role of *Trichoderma* in the soil and the use of native strains and/or their metabolites as a solution for protecting grapevine plants from GTDs. We isolated *Trichoderma* from the soil to protect the roots of grapevine plants while investigating factors that favor its presence in the vineyard. We found that the presence of *Trichoderma* is positively correlated with a high content of iron in the soil and negatively correlated with the pH value. Moreover, management practices affect *Trichoderma* soil populations and they are negatively correlated with fertilization and tillage but positively correlated with spraying herbicides in soils. We isolated 21 *Trichoderma* strains, and first evaluated them for their antibiosis activity. Great variability among strains was found even in the same soils. Two strains were selected (T065 and T087) as effective producers of secondary metabolites with significant activity against *P. minimum*. Later, a second mechanism of biocontrol was evaluated: mycoparasitism. In this case, the pathogen’s growth was reduced using the two previously selected *Trichoderma* strains (T065 and T087). Both are promising candidate biological control agents, especially T087, whose high production of spores indicates that it is an appropriate candidate for mass production and commercialization.

First, we collected soil samples from each vineyard with the aim of analyzing its physicochemical characteristics and isolating *Trichoderma* strains at the same time. In our study, the media used for *Trichoderma* isolation contains dextrose, rose bengal and chloramphenicol, which are suitable for *Trichoderma* species isolation [32]. Only 21 different isolates were found from ten different vineyards. In addition, we did not isolate *Trichoderma* spp. in two plots (ARE and TER). All of these plots are located in the inner plateau of Spain and the same climate is experienced by all of them, as described in a previous study of these locations [33]. However, the soil microbial community could also play an important role [34] and could drive different compositions of fungal and bacterial communities, as was demonstrated previously [35,36]. Other parameters such as the soil temperature, redox status of the soil and moisture could also play an important role [37]. In this study we identified a negative correlation with pH; the lower the pH, the higher the presence of *Trichoderma*. It has been demonstrated that the soil pH crucially influences the population of bacterial and fungal communities [35,37].This parameter is considered one of the major factors that affects the activity of *Trichoderma* and this fungus has been found to be better adapted to acidic soil [38]. Another study found that there is also a negative correlation between the pH and the activity of a *Trichoderma koningii* biological control strain in agricultural soils of wheat [39]. A recent study also found that some other biological control *Trichoderma* strains (T029 and T059) had a negative correlation with the pH in soils in comparison to its abundance in the soil in bean fields [40]. In our case, native *Trichoderma* populations in vineyards of Castilla y León are increased in acidic soils (pH between 5.5 and 6.5). We also observed a positive correlation between the abundance of *Trichoderma* and the iron content in soil. *Trichoderma* is able to produce organic acids that permit the solubilization of phosphates or micronutrients and mineral cations [38,41]. Several studies have concluded that *Trichoderma* strains (*Trichoderma asperellum* T34, *Trichoderma asperellum* T9) applied to the soil could increase the iron uptake of plants [42,43,44,45]. An example is harzianic acid, a novel siderophore produced by *Trichoderma harzianum* M10 that alters nutrient availability in soil due to the mechanism of iron solubilization [46]. In this study, this positive correlation could be due to a low pH that increased iron solubilization and the presence in a higher proportion of *Trichoderma* strains in natural soils that could increase the availability of iron in soil among other microorganisms [47]. This hypothesis could be contrasted and evaluated using a blue agar CAS assay for siderophore detection as possible future research [48]. Using factor analysis in this research with small size samples produces a reliable degree of confidence [49], but laboratory studies are needed to confirm both trends and the type of correlation. In this case, it is more important to unravel the type of correlation (causality or consequence) in these interactions to increase the *Trichoderma* population in soils. Moreover, a deeper analysis is required in order to obtain more insights about the relationships between *Trichoderma* and abiotic or biotic factors as described by Sorribas et al. 2008 [50]. Regarding farming management practices, spraying herbicides in vineyard rows had a positive correlation with the population of *Trichoderma* in our research. *Trichoderma* indigenous isolates from soil in India demonstrated a high compatibility with all the isolates in comparison to some pesticides. Some of them, such as 2,4-D and glyphosate, showed compatibility with a few strains [51]. Moreover, some *Trichoderma* strains such as *Trichoderma viride* strain FRP could degrade glyphosate [52]; *Trichoderma asperellum* TJ01 is tolerant to an organophosphorus pesticide [53]. *Trichoderma atroviride* UEL257 showed a higher tolerance to some of the herbicides tested and it was recommended to be sprayed in soils where there is a strong presence of herbicides [54]. Recent studies emphasize the possibility of using glyphosate-eating fungi such as *Trichoderma*, *Fusarium*, *Aspergillum* or *Penicillium* to tolerate this pesticide [55] and another study remarks the efficiency of glyphosate degradation by microorganisms such as *Penicillium*, *Aspergillus* and *Trichoderma* [56]. However, the use of herbicides sprayed in rows disrupts and reduces fungal communities and could lead to a reduction in crop production [13] so that native *Trichoderma* strains could develop a higher resistance to the pesticides sprayed in comparison to other fungi or bacteria, and thus could occupy this ecological niche due to its rapid growth and opportunism [57]. This hypothesis should be tested and evaluated to see if these isolates are able to resist different herbicides. Furthermore, no tillage was found to help increase the population of native *Trichoderma* strains. Tillage destroys fungal hyphae and reduces the presence of fungal communities [58]. Another study shows that non-tillage favors fungi and more diverse fungal communities [59]. In addition, in another study where the presence of *Trichoderma* was evaluated in comparison to practice management, a higher presence of this fungus was found when no tillage was performed in the field [19]. Finally, fertilization did not have a positive correlation with the presence of *Trichoderma* in soils evaluated. Several studies show that in fertilized soils there is an increase in the microbial biomass, crop yield and crop quality compared to non-fertilized soils [19,21,60,61]. An increase in the *Trichoderma* population was also found after applying fertilizers [62]. In this study, no differences in terms of the type of fertilization (manure or chemical fertilizers) were described to facilitate a reliable statistical analysis. As significant differences were achieved when applying any type of fertilization, it could also be due to an alteration of the ratio of carbon to nitrogen in the soil (C/N) or to other nutrients that alter the composition of the microbiota in soil [63]. To sum up, the highest presence of *Trichoderma* was found in acid soils (pH 5.5–6.5) where non-tillage, fertilization and herbicide spraying are performed. For future perspectives, further assays are needed to separately confirm the factor that has a crucial incidence in this interaction between factors.

In terms of biocontrol, for an eco-sustainable agriculture, *Trichoderma* is one of the most suitable biological control agents for this aim [4]. In this study, fungi of the *Trichoderma* genus were isolated in an attempt to reduce the incidence of fungi that cause GTDs through the roots. In this case, *Phaeoacremonium minimum* was used as a very aggressive pathogen that can attack the plant via pruning wounds but also in the roots [8]. In previous complementary studies, *Trichoderma* strains were isolated from grapevine bark and tested against *Phaeoacremonium minimum* with the aim of controlling infection in pruning wounds [33], and *Trichoderma* strains were selected to improve its performance—related to climatic factors [64]—on these pruning wounds. In our study we selected *Trichoderma* strains from the soil due to its capacity to produce secondary metabolites. Synthetic chemical pesticides have been used for years but their impact on human health and the environment has led to a change and a new approach to search for agriculturally important microorganisms such as *Trichoderma* [2]. Secondary metabolites of *Trichoderma* have different biological roles, some of them exhibiting direct activity against plant pathogens, but also triggering plant defenses or enhancing vegetal growth [65]. In our research, *in vitro* cellophane membrane assays led us to select the *Trichoderma* strains able to produce metabolites with antibiotic activity against *P. minimum*. We obtained two strains that significantly reduced the growth of this pathogen. The first was T065, identified as *T. gamsii* [31]. Another fungus of this species (*T. gamsii* strain ICC080), along with other *T. asperellum,* have been used as biocontrol agents of esca and grapevine trunk diseases, and they have been able to reduce GTD incidence and protect grapevine plants [66,67]. Secondly, T087 was identified as *T. harzianum*. In this case, *T. harzianum* and *T. atroviride* are the two species most widely used in the biocontrol of GTDs [10]. *T. harzianum* has been used for protecting vine cuttings (*T. harzianum* T39 Trichodex^®^) [68], and selected strains have been used to avoid the decline in pathogens in nursery grapevine plants using Trichoflow-T™ [69]. Molecular identification is an important factor in unravelling the great disparity of results in *Trichoderma* field assays and trying to fix the factors that govern each genotype’s function [70]. One of the first steps is to reach the strain level because even *Trichoderma* from the same species present differences [71]. Moreover, merely observing molecular identification may lead to some problems, especially in the *harzianum–virens* clade [72]. If the whole genome of some biological control agents is sequenced and its genes are identified, interesting new synthetic pathways and other relations can be found, as described by Schmoll et al. [73]. However, a final step is also necessary in order to confirm this effectiveness. It is important to test a biological control agent in a three-way interaction (*Trichoderma*–plant–pathogen) [7] before being sold on the market. Proteomic assays [74], microscopic assays [75] or metabolomic assays [76] must be conducted in planta to confirm a proper control of pathogens and to reduce the great disparity in results described previously. Moreover, other strains of *T. harzianum* have been described to protect plants from pruning-wound infections [75,77,78]. In addition, the application of a major secondary metabolite (6-pentyl-α-pyrone (6PP)) produced by *Trichoderma* strains (one of them was *T. harzianum* T77) against *Eutypa lata*, *Neofussicocum australe*, *Neofusiccocum parvum* and *Phaeomoniella chlamydospora* (pathogens involved in GTDs) triggered the inhibition of ascospore germination and a reduction in mycelial growth [79]. In our research, only diffusible antifungal compounds (DACs) have been evaluated. It could also be interesting to identify volatile organic compounds (VOCs), as has been described by Van Jaarsveld et al. [80] against black foot disease pathogens. Our future work will evaluate the biocontrol mechanism of mycoparasitism, which would most likely be one of the ancestral lifestyles of *Trichoderma* [81]. We assayed our selected *Trichoderma* strains (T065 and T087) against *P. minimum* in a dual-culture assay and saw that both of them stopped the growth of this pathogen, as in another study described by Carro-Huerga et al. [33] where native *Trichoderma* strains isolated from the bark of grapevine plants could mycoparasitize *P. minimum.* In dual-confrontation assays two types of mechanisms are used: antibiosis (as previously assayed in the membrane growth assay in this research) [82] and mycoparasitism [83,84]. Therefore, we confirmed the biocontrol by performing an *in vitro* test of two strains (T065 and T087). T087 could especially be a potential microorganism for mass production due to its capacity for high production spores, as most *Trichoderma*-based products are commercialized in spores [6].

To sum up, our results indicate that (i) physicochemical soil properties and farming management practices affect native *Trichoderma* populations and (ii) native *Trichoderma* strains isolated from vineyard soil biocontrol *P. minimum*. According to the former, the pH and iron content of soils are correlated to *Trichoderma* abundance and farming management practices also affect its presence. Regarding the second hypothesis, two *Trichoderma* strains (T065 *Trichoderma gamsii* and T087 *Trichoderma harzianum*) exhibited a significant biocontrol activity against *P. minimum* using antibiosis as the main mechanism. Future assays should evaluate siderophore production of the isolated *Trichoderma* strains, test the resistance to biodegrade herbicides, analyze secondary metabolite profiles of both *Trichoderma* strains selected and determine their major compounds to evaluate the suitability of their application in field.

## 4. Materials and Methods

### 4.1. Study Sites and Sampling

Vineyards located in 4 Spanish wine-producing regions belonging to Castilla y León (Spain) were chosen for soil sampling. These plots were placed in municipal districts of Cacabelos (42°35′59″ N 6°43′32″ W), Peñafiel (41°35′51″ N 4°07′22″ W), El Pego (41°19′59″ N 5°28′09″ W), Gordoncillo (42°08′03″ N 5°24′10″ W), La Antigua (42°10′45″ N 5°41′22″ W) and La Bañeza (42°17′51″ N 5°54′06″ W), the same locations where samples of bark from plants were also taken as part of another study for *Trichoderma* isolation [33]. They were coded with three/four capital letters and their main characteristics are shown in Table 8. In total, ten plots were surveyed, and they belong to a Protected Designation of Origin (PDO). PDO is a certification to distinguish quality schemes for agricultural products and foodstuffs of a specific region (EC Reg. n. 1493/1999. 8 August 2009, OJC 187).

Soil sampling was undertaken during the winter season (between the end of January and end of February) and was performed as follows. Five cores were taken randomly in a zigzag under the crop line using a 9 mm diameter soil auger to a depth of 10–30 cm in each area (ca. 200 g/sample, total of 10 samples). Each sample was kept in a clean plastic container. After that, they were air dried in a laboratory for one week by spreading each sample on a tray to air dry at room temperature and sieved (2 mm mesh size) prior to soil physicochemical analyses and *Trichoderma* isolation.

### 4.2. Farming Management Practices

Viticulturists were surveyed according to soil management practices during the last five campaigns. Tillage on rows, herbicides on rows and fertilization were fixed as parameters that could influence the *Trichoderma* population.

### 4.3. Soil Processing

#### 4.3.1. Soil Physicochemical Analysis

Half of each sample from the soil (after drying and sieving) was sent to the Laboratory of Instrumental Techniques of the University of León (León, Spain) for further analysis according to the official methods of the Spanish Ministry of Agriculture [85]. Briefly, pH was measured in a soil:water suspension in a 1:2.5 ratio and subsequent read with a potentiometric method using a pH meter. Textural class was determined using the Bouyoucos densimeter method [86]. The total N was analyzed using the Kjeldahl method [87]. For the soil organic matter (SOM%), the Walkley Black method [88] was used. The Olsen method was used for the determination of assimilable phosphorus (P) [89].

Cations such as calcium (Ca), magnesium (Mg) and potassium (K) were extracted with an AcONH4 1N pH 7 solution and subsequently read with inductively coupled plasma optical emission spectrometry (ICP-OES 5110 SVDV, Agilent Technologies, Victoria, Australia and autosampler Agilent SPS 4,Tokyo, Japan). In the determination of cation exchange capacity (CEC), a 0.1 M barium chloride solution and reading with ICP-OES was used. As for trace elements such as manganese (Mn), iron (Fe), copper (Cu) and zinc (Zn), these were extracted with a diethylen triamine pentaacetic acid (DTPA) pH 7.3 solution and read using ICP-OES. Finally, boron (B) extraction was realized with hot water and using ICP-OES.

#### 4.3.2. *Trichoderma* Isolation and Quantification

The other half of each soil sample was used for *Trichoderma* isolation. Methodology for the extraction of fungal isolates from soil was used according to [90]. Briefly, 5 g of soil was added to 45 mL of 1% hydroxyethyl cellulose (Sigma-Aldrich Chemie GmbH, Steinheim, Germany), after that, they were placed in mechanical agitation (mechanically shaken) for one hour. Later, 10-fold serial dilutions were made, and subsequently 100 µL of each dilution was plated in triplicate onto semi-selective rose bengal–chloramphenicol agar medium (Sigma-Aldrich Chemie GmbH, Steinheim, Germany). The quantity of *Trichoderma* spp. in each soil was assorted in a quantitative table of values for comparison with other variables. Values of *Trichoderma* soil abundance from 0 to 4 were assigned to each soil. These values were related to the presence of any isolate of *Trichoderma* in the lowest concentration for each serial dilution as follows: 10^−3^ dilution = 4; 10^−2^ dilution = 3; 10^−1^ dilution = 2; 1 = 1; none = 0. Thus, the larger the presence in a sample related to the serial dilutions, the higher the value assigned to its presence. When there were no *Trichoderma*, a zero was assigned to its value. Isolates were identified based on their morphology according to [30].

#### 4.3.3. Identification of Parameters Involved in *Trichoderma* spp. Abundance

Statistical analysis was performed in order to establish a correlation between (1) the analysis of physicochemical properties of soil and *Trichoderma* soil abundance and (2) the management regime and *Trichoderma* soil abundance.

Physicochemical properties and *Trichoderma* soil abundance data were evaluated to establish whether they had a normal distribution and no collinearity to perform a principal component analysis (PCA).

In addition, the management regime and *Trichodema* soil abundance were evaluated to establish whether they had a normal distribution and no collinearity to perform a multiple factor analysis (MFA) due to the categorical and numerical values shown.

R software (R Core Team, 2013) was used. To carry out PCA and MFA, the FactoMineR package—a graphical user interface [91] was used. In this study, five dimensions were fixed by default. Consequently, the eigenvalues of *Trichoderma* were obtained and compared to the rest of the values in those dimensions that were also significant according to a *p* value < 0.05. Finally, a plot of these values was created to justify the conclusions.

### 4.4. DNA Extraction, PCR Amplification and Sequencing of Trichoderma Strains

Genomic DNA was isolated from 100 mg of mycelia of each fungal isolate. The manufacturer’s protocol for fungi of the Nucleospin Plant II kit (Macherey-Nagel, Düren, Germany) was performed. Extracts were diluted in 50 μL of sterile water. A NanoDrop ND-1000 spectrophotometer (Thermo Scientific, Wilmington, DE) was used to estimate the DNA concentration. Amplifications were performed using 50 ng of template DNA in a final volume of 50 μL containing 10 mM Tris–HCl (pH 8.3), 50 mM KCl, 1.5 mM MgCl2, 200 M for each dNTP, 400 nM for each primer and 1.5 U of DreamTaq DNA polymerase (Thermo Scientific, Wilmington, DE). In total, 21 isolates were selected for analyzing the primer pair ITS5 (5′-GGAAGTAAAAGTCGTAACAAGG-3′) and ITS4 (5′-TCCTCCGCTTATTGATATGC-3′) that was used to amplify nuclear rDNA ITS regions [92]. The PCR products were first purified with the Nucleo Spin Extract II kit (Machery-Nagel, Düren, Germany) and were then sequenced using the primer pair ITS4–ITS5, the kit BigDye Terminator v3.1 Cycle Sequencing Kit (Applied Biosystems, Foster City, CA) and the automatic capillary sequencer ABI 3130xl (Applied Biosystems) according to the manufacturer’s instructions. Sequences were introduced in databases such as the NCBI Genbank (National Center for Biotechnology Information, http://www.ncbi.nlm.nih.gov) using the BLAST program (http://www.ncbi.nlm.nih.gov/BLAST; accessed on 14 September 2022) to identify the fungi. In addition, the EF1-α gene was amplified using the primers EF1-728F (5′-CATCGAGAAGTTCGAGAAGG-3′) and EF1-986R (5′ TACTTGAAGGAACCCTTACC-3′) [93] for the isolate T087 and it was identified as previously described. Another *Trichoderma* selected (T065) was identified previously in [31].

### 4.5. In Vitro Antifungal Assays of Trichoderma Strains against Phaeoacremonium minimum

#### 4.5.1. Growth Membrane Assays

To evaluate the inhibitory activity of the *Trichoderma* isolates’ secretome, a cellophane membrane was used in order to test it as previously described in [94] with some modifications. The pathogen *Phaeoacremonium minimum* was provided by the Instituto Tecnológico Agrario de Castilla y León (ITACyL) Y038-05-03a and was described as an aggressive isolate in grapevine plants [95].

**Figure 6:**
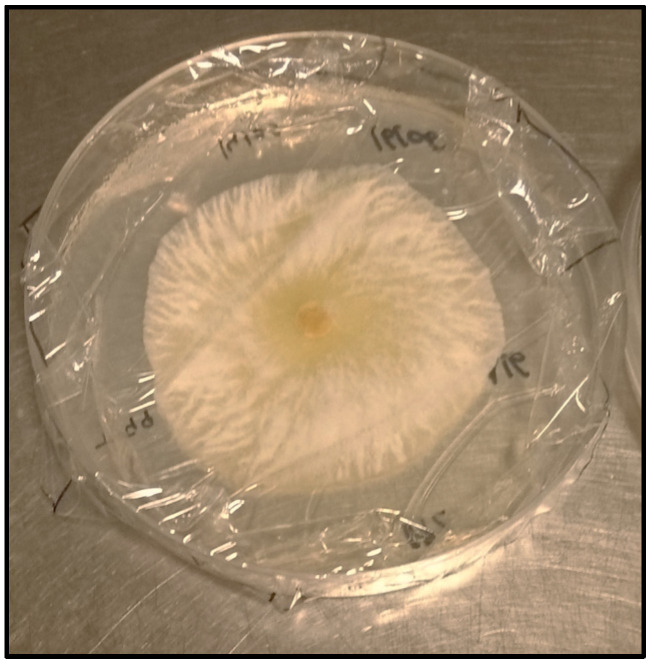
*Trichoderma* T099 after 2 days over cellophane membrane and PDA medium.

In short, Petri dishes of PDA medium were overlaid with a sterile cellophane membrane. *Trichoderma* plugs extracted from PDA dishes grown for 7 days at 22 °C, were placed in the center of the Petri dish with the cellophane membrane containing PDA medium and incubated for 48 h at 22 °C (Figure 6). Then the cellophane membranes along with the mycelia of each *Trichoderma* isolate were removed and *P. minimum* plugs were placed in the same plates after previously being incubated for 15 days at 22 °C. The growth of *P. minimum* was recorded after 14 days to calculate the percentage of pathogen growth inhibition. In addition, control plates were performed where pathogen *P. minimum* was placed in order to compare the different treatments, as described above. Growth inhibition assays were performed in quadruplicate and the results are expressed as the percentage of *P. minimum* growth inhibition by each *Trichoderma* strain tested. SPSS software (Statistics for Windows Version 26.0, IBM Corp., Armonk, NY, USA) was used for all statistical analyses.

#### 4.5.2. Dual-Confrontation Assays

*Trichoderma* strains before being selected (T065 and T087) were chosen to perform a dual-confrontation assay against *P. minimum* as previously described [33]. The culture of the pathogen alone was used as control. The colony area of *P. minimum* was measured after 5 days of co-inoculation with *Trichoderma* strains at 22 °C in the dark. Moreover, morphological and phenotypic behavior was recorded, such as sporulation on plate, sporulation on pathogen and the production of yellow pigment. For each assay, four repetitions for each *Trichoderma* were performed and the results are expressed as the growth inhibition percentage of the *P. minimum* colony.

#### 4.5.3. Statistical Analysis

In both cases, growth membrane assays and dual-confrontation assays were analyzed using IBM SPSS^®^ statistics 26 (IBM Corp.). This computer program was used for the statistical analyses as follows: first, it checked if they had a normal distribution using Shapiro–Wilk tests, then the homogeneity of variances was evaluated using Levene’s test and one-way ANOVAs were carried out to determine if there were significant differences. A post hoc test (Duncan, *p* < 0.05) was performed to establish differences between groups.

## 5. Conclusions

Despite a wide use of *Trichoderma* in viticulture for preventing GTDs, little is known about the interactions between *Trichoderma* strains and the environment. Overall, this research showed a correlation between physicochemical parameters, farming management and *Trichoderma* abundance in vineyard soils of Castilla y León. Moreover, it found two native *Trichoderma* strains (T065 *Trichoderma gamsii* and T087 *Trichoderma harzianum*) isolated from vineyards in Castilla y León region that possess antibiotic and mycoparasitic activity against *P. minimum*, one of the fungi that are involved in GTDs. Finally, it proved the potential that native strains have in terms of biocontrol of pest and diseases.

## Figures and Tables

**Table 1:** Management regime of the ten vineyard soils examined in this study.

Plot	Tillage	Herbicides	Fertilization
Protected Designation of Origin Leon			
PLC (La Antigua, Castilla y León)	yes	no	yes
RIB (La Bañeza, Castilla y León)	yes	no	yes
CAL (Gordoncillo, Castilla y León)	yes	yes	yes
MAZ (Gordoncillo, Castilla y León)	yes	yes	yes
Protected Designation of Origin Toro			
PEGB (El Pego, Castilla y León)	yes	no	yes
Protected Designation of Origin Bierzo			
GLM (Cacabelos, Castilla y León)	no	yes	no
GLG (Cacabelos, Castilla y León)	no	yes	no
GVG (Cacabelos, Castilla y León)	no	yes	no
Protected Designation of Origin Ribera del Duero			
ARE (Peñafiel, Castilla y León)	yes	no	yes
TER (Peñafiel, Castilla y León)	yes	no	yes

**Table 2:** Physicochemical characteristics of soil samples analyzed in this study.

	PDO León				PDO Toro
***Plot***	PLC	RIB	CAL	MAZ	PEGB
***Location***	La Antigua	La Bañeza	Gordoncillo	Gordoncillo	El Pego
***pH***	5.91	8.13	6.92	6.26	5.12
***Clay (%)***	4.00	10.00	16.00	22.00	10.00
***Sand (%)***	66.00	38.00	56.00	50.00	86.00
***Silt (%)***	30.00	52.00	28.00	28.00	4.00
***SOM (%)***	1.66	0.91	1.05	1.11	0.44
***Total N (%)***	0.12	0.15	0.10	0.08	0.04
***P (cmol(+)kg^−1^)***	5.44	87.93	13.31	6.77	22.15
***Ca (cmol(+)kg^−1^)***	2.20	21.51	2.73	3.63	4.22
***Mg (cmol(+)kg^−1^)***	0.68	0.50	0.68	0.89	1.22
***K (cmol(+)kg^−1^)***	0.40	0.85	0.40	0.36	0.29
***Mn (mg/1000 g)***	28.18	6.88	46.02	60.88	37.10
***Fe (mg/1000 g)***	14.27	9.15	14.51	14.09	17.61
***Cu (mg/1000 g)***	0.44	2.76	1.12	1.00	0.89
***Zn (mg/1000 g)***	0.86	1.98	1.00	0.57	0.18
***B (mg/1000 g)***	0.27	0.62	0.73	1.05	0.15
	**PDO Bierzo**			**PDO Ribera del Duero**	
***Plot***	GLM	GLG	GVG	ARE	TER
***Location***	Cacabelos	Cacabelos	Cacabelos	Peñafiel	Peñafiel
***pH***	5.88	6.25	5.50	8.10	8.26
***Clay (%)***	18.00	18.00	16.00	10.00	20.00
***Sand (%)***	32.00	28.00	30.00	70.00	50.00
***Silt (%)***	50.00	54.00	54.00	20.00	30.00
***SOM (%)***	2.20	1.56	0.91	0.97	1.60
***Total N (%)***	0.26	0.17	0.14	0.08	0.06
***P (cmol(+)kg^−1^)***	10.54	5.44	9.90	31.12	12.99
***Ca (cmol(+)kg^−1^)***	5.11	3.77	2.06	18.73	18.58
***Mg (cmol(+)kg^−1^)***	1.79	1.07	0.38	0.86	2.41
***K (cmol(+)kg^−1^)***	0.51	0.50	0.71	0.67	0.39
***Mn (mg/1000 g)***	41.65	35.27	30.09	7.57	3.94
***Fe (mg/1000 g)***	13.11	10.91	12.99	4.98	3.11
***Cu (mg/1000 g)***	16.50	15.16	7.99	1.71	1.12
***Zn (mg/1000 g)***	1.73	1.43	2.20	2.36	1.34
***B (mg/1000 g)***	0.32	0.36	0.41	0.42	0.19

The results were obtained following the procedure described in Section 4.3.1, Soil physicochemical analysis. Variables measured: pH of soil; percentage of clay, sand and silt, soil organic matter (SOM); nitrogen (total N); assimilable phosphorus (P); the cations potassium (K), calcium (Ca) and magnesium (Mg); and the microelements manganese (Mn), iron (Fe), copper (Cu), zinc (Zn) and boron (B). PDO—Protected Designation of Origin.

**Table 3:** Principal components analysis (PCA) of soil values. Eigenvalues, cumulative variance of the dimension loadings (significant in bold) between the dimensions and the characters studied.

Soil Parameters ^1^	Dim1	Dim2	Dim3
Sand	−0.932	−0.2473	−0.062
Silt	**0.9478**	0.1958	−0.0923
Clay	0.3418	0.2573	0.4881
pH	0.2335	**−0.8223**	0.0502
SOM	0.5386	0.1691	**0.6546**
Total N	**0.7985**	0.3461	0.0785
Mg	−0.0237	−0.2212	**0.9532**
K	**0.6679**	−0.4206	−0.5811
Fe	−0.3605	**0.8345**	−0.2705
Zn	**0.751**	−0.5131	−0.2523
B	0.1168	0.2756	−0.3831
*Trichoderma*_soil_abundance	0.2857	**0.8761**	−0.0583

^1^ 77.49 % of the information (variance) contained in the data are retained by the first three principal components. Soil physicochemical analysis parameters: pH of soil; percentage of clay, sand and silt; soil organic matter (SOM); nitrogen (Total N); assimilable phosphorus (P); the cations potassium (K), calcium (Ca) and magnesium (Mg); and the microelements manganese (Mn), iron (Fe), copper (Cu), zinc (Zn) and boron (B).

**Table 4:** Comparing all values to *Trichoderma* soil abundance; there is a significant correlation between the following parameters *p* < (0.05) in dimension 2.

	Correlation	*p* Value
Fe	0.8345	0.0027
pH	−0.8223	0.0035
*Trichoderma*_soil_abundance	0.8761	0.0009

pH (pH of soil); Fe (iron of soil).

**Table 5:** Multiple factor analysis (MFA) management: eigenvalues, cumulative variance of the dimension loadings (significant in bold) between the dimensions and the characters studied.

Management	Dim.1	Dim.2	Dim.3
*Trichoderma* soil abundance	**0.7903**	0.5379	0.2936
Tillage	**0.9207**	−0.3875	0.0469
Herbicides	**0.8694**	0.3318	−0.3663
Fertilization	**0.9207**	−0.3875	0.0469

100.00% of the information (variance) contained in the data are retained by the first three main components.

**Table 6:** Comparing all values to *Trichoderma* soil abundance: there is a significant correlation of the following parameters *p* < (0.05) in dimension 1.

Quantitative Parameters		
Values	correlation	*p* value
*Trichoderma* soil abundance	0.7903	0.0065
Qualitative parameters		
Values	R^2^	*p* value
Tillage.in.lines	0.8477	0.0002
fertilization	0.8477	0.0002
herbicides.in.line	0.7558	0.0011
*Category of evaluation*		
Values	Estimate	*p* value
fertilization = fertilization_no	1.7617	0.0002
Tillage.in.lines = Tillage.in.lines_no	1.7617	0.0002
herbicides.in.line = herbicides.in.line_yes	1.5246	0.0011
herbicides.in.line = herbicides.in.line_no	−1.5246	0.0011
fertilization = fertilization_yes	−1.7617	0.0002
Tillage.in.lines = Tillage.in.lines_yes	−1.7617	0.0002

**Table 7:** Dual-culture assay of *Trichoderma* spp. isolates selected (T065 and T087) after 5 days at 25 °C with the plant pathogenic fungus *P. minimum* on PDA.

Strain	Dual-Culture (%) ^1^	Sporulation on Plate ^2^	Sporulation on Pathogen ^2^	Production of Yellow Pigment ^2^
T065	15.52 ± 2.23 a	0.00	0.00	0.00
T087	15.00 ± 1.83 a	1.00	0.00	0.00

^1^ Results are expressed as the inhibition percentage compared to the control *P. minimum* grown alone. Values are means of four replicates. The letters indicate means between which there are no statistically significant differences (*p* = 0.05), according to the Duncan post hoc test applied to normalized data. ^2^ Morphological characteristics (sporulation on plate, sporulation on pathogen and production of yellow pigment) were assessed using a 0 to 3 scale in which the values were coded as follows: 0, absence; 1, weak; 2, heavy; and 3, very heavy.

**Table 8:** Site, location, date sampled and year vineyard established of the studied soils.

Nomenclature of Place ^1^	Location	Dates Sampled	Year Vineyard Established
PDO LEON			
Site PLC (Pago Laguna del Caballo)	La Antigua, Castilla y León	January 2016	1934
Site RIB (Ribas de La Valduerna)	La Bañeza, Castilla y León	January 2017	2001
Site CAL (Calabazanos)	Gordoncillo, Castilla y León	February 2017	2006
Site MAZ (Casa Mazo)	Gordoncillo, Castilla y León	February 2017	1997
PDO TORO			
Site PEGB (El Pego)	El Pego, Castilla y León	February 2016	1926
PDO BIERZO			
Site GLM (Legua I)	Cacabelos, Castilla y León	January 2017	1995
Site GLG (Legua II)	Cacabelos, Castilla y León	January 2017	2011
Site GVG (Legua III)	Cacabelos, Castilla y León	January 2017	1937
PDO RIBERA DEL DUERO			
Site ARE (Arenosas)	Peñafiel, Castilla y León	February 2017	2001
Site TER (Terrazas)	Peñafiel, Castilla y León	February 2017	2008

^1^ Same locations as part of another study [33].

## Data Availability

Not applicable.

## Supplementary Materials

The following supporting information can be downloaded at: https://www.mdpi.com/article/10.3390/plants12040887/s1, Figure S1: Potato dextrose agar (PDA) cultures after 7 days of the 21 *Trichoderma* isolates that were obtained in this study.
