# Supplementary material for: Vineyard Management and Physicochemical Parameters of Soil Affect Native Trichoderma Populations, Sources of Biocontrol Agents against Phaeoacremonium minimum
Source: Plants (Basel). 2023 Feb 16;12(4):887. doi: 10.3390/plants12040887 (PMC9966749; doi:10.3390/plants12040887)
Supplement: Supplementary file 1 [file plants-12-00887-s001.zip › plants-2203009-supplementary.pdf]

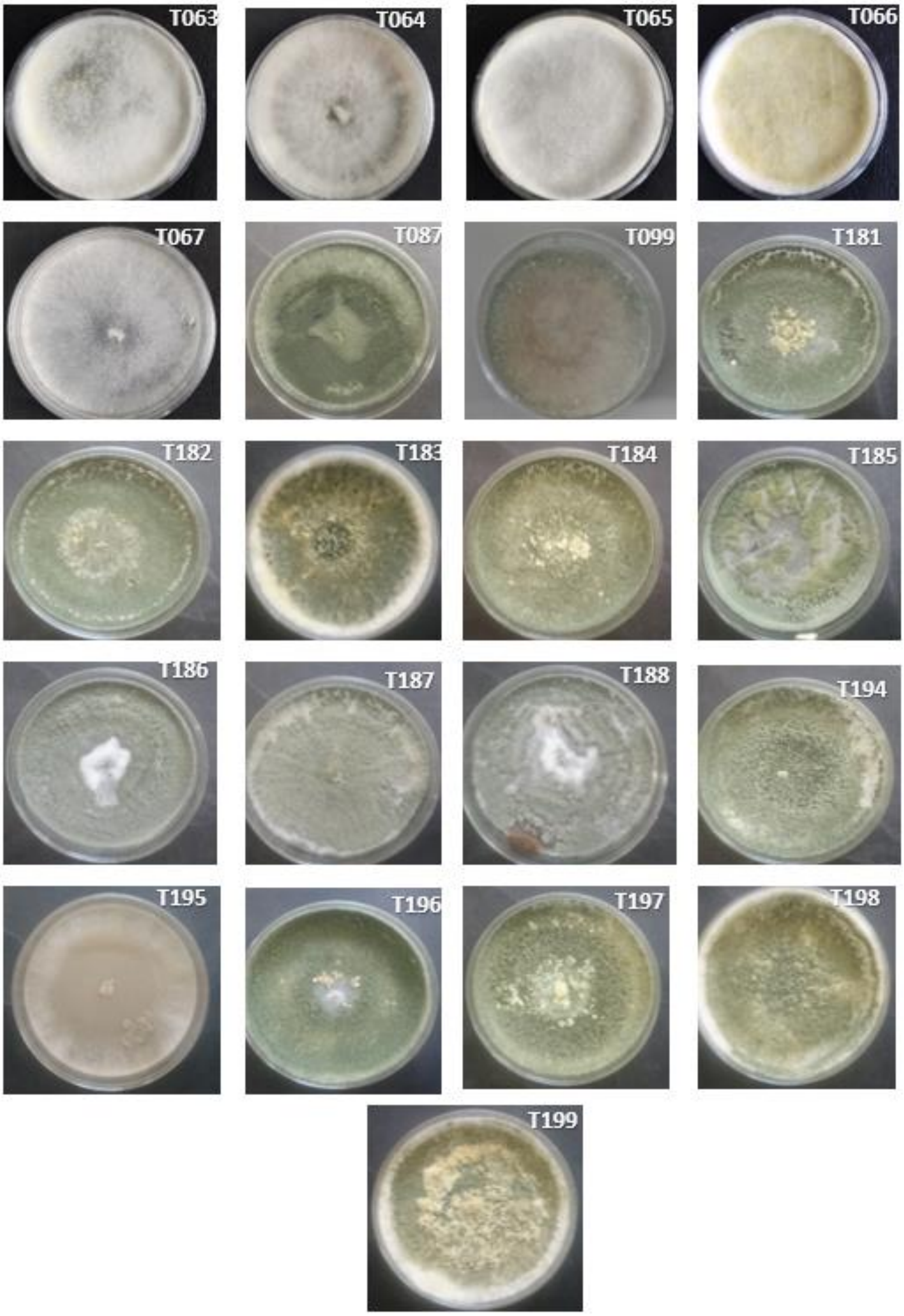

Figure S1. Potato dextrose agar (PDA) cultures after 7 days of the 21 *Trichoderma* isolates that were obtained in this study.
